# IDO and galectin-3 hamper the ex vivo generation of clinical grade tumor-specific T cells for adoptive cell therapy in metastatic melanoma

**DOI:** 10.1007/s00262-017-1995-x

**Published:** 2017-04-11

**Authors:** Sara M. Melief, Marten Visser, Sjoerd H. van der Burg, Els M. E. Verdegaal

**Affiliations:** 1 Department of Medical Oncology, Leiden University Medical Center, Leiden, The Netherlands; 2 Department of Clinical Oncology, Leiden University Medical Center, P.O. Box 9600, 2300 RC Leiden, The Netherlands

**Keywords:** Metastatic melanoma, Adoptive cell transfer, Immunotherapy, GMP, IDO, Galectin-3

## Abstract

Adoptive T cell transfer (ACT) with ex vivo-expanded tumor-reactive T cells proved to be successful for the treatment of metastatic melanoma patients. Mixed lymphocyte tumor cell cultures (MLTC) can be used to generate tumor-specific T cells for ACT; however, in a number of cases tumor-reactive T cell, expansion is far from optimal. We hypothesized that this is due to tumor intrinsic and extrinsic factors and aimed to identify and manipulate these factors so to optimize our clinical, GMP-compliant MLTC protocol. We found that the tumor cell produced IDO and/or galectin-3, and the accumulation of CD4^+^CD25^hi^FoxP3^+^ T cells suppressed the expansion of tumor-specific T cells in the MLTC. Strategies to eliminate CD4^+^CD25^hi^FoxP3^+^ T cells during culture required the depletion of the whole CD4^+^ T cell population and were found to be undesirable. Blocking of IDO and galectin-3 was feasible and resulted in improved efficiency of the MLTC. Implementation of these findings in clinical protocols for ex vivo expansion of tumor-reactive T cells holds promise for an increased therapeutic potential of adoptive cell transfer treatments with tumor-specific T cells.

**Electronic supplementary material:**

The online version of this article (doi:10.1007/s00262-017-1995-x) contains supplementary material, which is available to authorized users.

## Introduction

Melanoma, the most aggressive form of skin cancer, has long been recognized as a highly immunogenic tumor and a good target for immunotherapy [1]. High densities of different types of infiltrating immune cells have been correlated with increased (e.g., CD8 T cells) or worse (e.g., regulatory T cells) survival [2, 3]. Infusion of ex vivo-expanded tumor-reactive T cells resulted in objective tumor responses in metastatic melanoma patients [4, 5]. We showed that generation of T cell batches for infusion by weekly stimulation of peripheral blood mononuclear cells (PBMC)-derived T cells in mixed lymphocyte autologous tumor cell cultures (MLTC) leads to the expansion of high numbers of tumor-reactive T cells [5]. In depth, analysis of these tumor-reactive T cells showed that in selected cultures up to 80% of CD8 T cells highly selectively recognize autologous tumors and show very limited reactivity against HLA-matched melanoma cell lines and known shared antigens [6, 7]. However, for a number of patients, multiple MLTC were needed to obtain the required amount of T cells for infusion, suggesting that one or more tumor intrinsic or extrinsic factors negatively affect the activation of tumor-specific T cells [5].

Several tumor cell intrinsic factors have been found to hamper T cell immunity, including the expression of checkpoint receptors, the expression and secretion of galectins, or indoleamine 2,3-dioxygenase (IDO) [8–11]. The immunosuppressive effects of galectin-3 and galectin-1 are most often described [12–14]. Galectin-1 induces the differentiation of immunoregulatory, IL-10 producing Th cells [15], whereas galectin-3 can hamper T cell activation by interfering with T-cell receptor signaling [16] and by binding to the checkpoint inhibitor LAG-3 [17]. Additionally, both galectin-1 and galectin-9 have been shown to increase apoptosis in T cells [18–21].

IDO is a well-known tumor-expressed molecule involved in immunosuppression and tolerance [9]. It catalyzes the rate-limiting first step in tryptophan catabolism [22] and thereby directly affects T cell proliferation due to tryptophan starvation [23]. In addition, the metabolites of tryptophan have a toxic effect on T cells. Furthermore, IDO may induce and sustain regulatory T cells (Tregs) [24, 25], which in turn may hamper efficient generation of tumor-reactive T cells [26, 27]. In the human setting, Tregs have been shown to suppress the expansion of tumor-associated antigen-specific T cells [27, 28] even in a melanoma antigen-specific manner [29, 30].

The objective of this study was to identify factors negatively affecting the expansion of tumor-reactive T cells and means to neutralize their effect in our clinical, GMP-compliant protocol to generate tumor-reactive T cells for use in adoptive cell transfer (ACT). Here, we show that co-expansion of CD4^+^CD25^hi^FoxP3^+^ T cells and/or tumor cell produced IDO and galectin-3 during MLTC have a negative impact on the activation and expansion of tumor-specific T cells, and we describe different manners to circumvent this.

## Materials and methods

### Mixed lymphocyte tumor cultures

Tumor-reactive T cells were generated using mixed lymphocyte tumor cultures (MLTC) using a protocol that was successfully applied previously for generation of tumor-reactive T cells [7, 31]. In brief, cryopreserved PBMC were thawed and co-cultured with irradiated, autologous tumor cells in a ratio of [10:1] in Iscove’s MDM (Lonza, Verviers, Belgium) supplemented with 8% heat-inactivated pooled human serum (Sanquin Bloodbank), l-glutamine (4 mM), penicillin (50 U/ml), and streptomycin (50 µg/ml) (all GMP-grade from Lonza). At day 0 of the MLTC, human recombinant IL-4 (5 ng/ml, Cellgenix) was added. Human recombinant IL-2 (Aldesleukin, Novartis) was added starting at day 2 (150 IU/ml) and every 2–3 days, half of the culture medium was refreshed with T cell medium containing IL-2. The T cells were weekly counted and restimulated using irradiated autologous tumor cells. After 4 weeks of culture, the T cell batches were tested for reactivity against the tumor by overnight stimulation of T cells with tumor cells. Thereafter, culture supernatant of the co-cultures was collected and stored at −20 °C until further use, and the T cells were stained for flowcytometric analysis. The IFNγ concentration in the culture supernatant was quantified by ELISA (Sanquin).

In some experiments, CD4^+^ T cells were depleted from the PBMC at the start of MLTC, or CD25^+^ cells were depleted from the cultured T cells at week 2 of the MLTC. Depletion of either CD4^+^ or CD25^+^ cells was done by MACS using CD4 or CD25 human microbeads (Miltenyi Biotec GmbH), followed by MACS separation according to the manufacturer’s recommendations using either LD columns (CD4) or LS columns (CD25).

To investigate the role of IDO in the induction of tumor-reactive T cells, the IDO inhibitor 1-methyl-tryptophan (1-MT) was added to several MLTC. Two different isoforms of 1-MT, 1-MT-L, and 1-MT-D (both from Sigma Aldrich), were used, both in a concentration of 150 µM. Also, l-tryptophan (150 μM; Sigma Aldrich) was added in some cultures, to control for the tryptophan depletion caused by IDO (due to low cell numbers, we were only able to test this for four tumor cell lines).

### Short inhibition assay

To test the capacity of the tumor cell lines to inhibit T cell activation and proliferation, a short inhibition assay was set up. PHA-stimulated (0.5 μg/ml), allogeneic PBMC were co-cultured with tumor cells in various ratios. In addition, the capacity of tumor-derived cell culture supernatants (TSN) to inhibit PBMC activation was investigated. To this end, tumor cells were plated in similar concentrations as was done for the co-culture. After 24 h, TSN was collected and added to PHA-stimulated PBMC. T cell activation was analyzed after 48 h by flowcytometric analysis for CD25 expression and by measuring the IFNγ concentration in the TSN. Using these data, the inhibition index was calculated using the formula: 100 − (%CD25^+^ [PBMC:tumor]/%CD25^+^ [PBMC] × 100). For assessment of proliferation, PBMC were stimulated in the presence of tumor cells for 5 days and at day 4 ^3^[H]-thymidine was added to the co-culture. Incorporation of ^3^[H]-thymidine was measured 16 h later. Some experiments were performed in the presence of the lectin-inhibitor *N*-acetyl-d-lactosamine (5 mM) (LacNAc; Sigma Aldrich), that blocks the activity of galectins.

### Flowcytometry

After incubation with LIVE/DEAD^®^ Fixable Yellow Dead Cell Stain (Molecular probes, Life Technologies), the cells that were tested for reactivity against the autologous tumor cell line were stained with CD3-V450, CD4-FITC, CD8-APCCy7, CD137-APC, CD154-PECF495, IFNγ-Alexa Fluor700, and IL-2-PE [where appropriate Brefeldin-A (10 µg/ml, Sigma Aldrich)] and were added to the cultures to perform intracellular staining. For assessment of regulatory T cell frequencies in the T cell batches, the cells were stained with CD3-V500, CD4-AlexaFluor700, CD8-FITC, CD25-PECy7, and FoxP3-PECF594 in combination with the Treg fixation/permeabilization kit (BD Biosciences). The activation of T cells in the short inhibition assay was determined by staining with CD3-Pacific Blue, CD8-PE, CD4-Alexa Fluor700, and CD25-PECy7. Tumor cells were assayed for galectin-3 expression by staining with galectin-3-Alexa Fluor647. All antibodies used for flowcytmetric analyses were from BD Biosciences (San Diego, USA). All flowcytometric analyses were performed using a BDLSRFortessa (BD Biosciences) and data analysis was done using FlowJo software (FlowJo, LLC).

### Analysis of IDO expression and enzymatic activity

For analysis of IDO expression and activity, irradiated tumor cells were cultured for 48 h in the absence and presence of 150 IU/ml IFNγ. Thereafter, the supernatant was collected and stored at −20 °C and the cells were harvested; the cell pellet was snap frozen and stored at −80 °C until further use. mRNA was extracted from the frozen cell pellets using the RNeasy plus mini kit (Qiagen N.V.) and cDNA was synthesized with the High capacity RNA-to-cDNA kit (Life Technologies Europe BV). RT-qPCR analysis of IDO expression (primerset *tcatctcacagaccacaa*/ *gcagtaaggaacagcaata*) was performed using IQ SYBR Green supermix (BioRad) on a CFX96 real-Time PCR detection system (Biorad). All RT-qPCR data were normalized to GAPDH (primerset *gtgctgagtatgtcgtggagtctac*/*ggcggagatgatgacccttttgg*) expression and analyzed using the delta-Ct method. In the culture supernatant, the concentration of kynurenine was measured as an indicator for the enzymatic activity of IDO as described before [32].

### Depletion of galectin-3 from TSN

To assess specifically the role of tumor-derived galectin-3 in the inhibition of T cell activation by tumor cells, we depleted galectin-3 from TSN that was used for the short inhibition assay. To this end, ELISA plates were coated with anti-galectin-3 antibodies (Peprotech). TSN was added to the coated ELISA plates and galectin-3 was depleted by serial incubation (4×) of the TSN in anti-galectin-3 coated wells for 2 h per incubation step. The efficiency of depletion was tested by a galectin-3 ELISA according to the manufacturers’ recommendations (Peprotech).

### Generation of galectin-3 knock-out cell lines using CRISPR/Cas9 genome editing

Cloning of the Cas9 target sequence was performed based on PCR and Gibson assembly (In-Fusion kit, Clontech Laboratories Inc.). Cells were transfected with a CRISPR-Cas9 construct targeting exon 3 of the human *galectin-3* gene. Transfections were performed using Lipofectamine^®^ 2000 (Thermofisher Scientific) according to manufacturers’ recommendations. Transfected cells were tested for surface expression as well as secretion of galectin-3.

## Results

### Accumulation of CD4^+^CD25^hi^FoxP3^+^ T cells during culture is associated with low T cell expansion

**Fig. 1:**
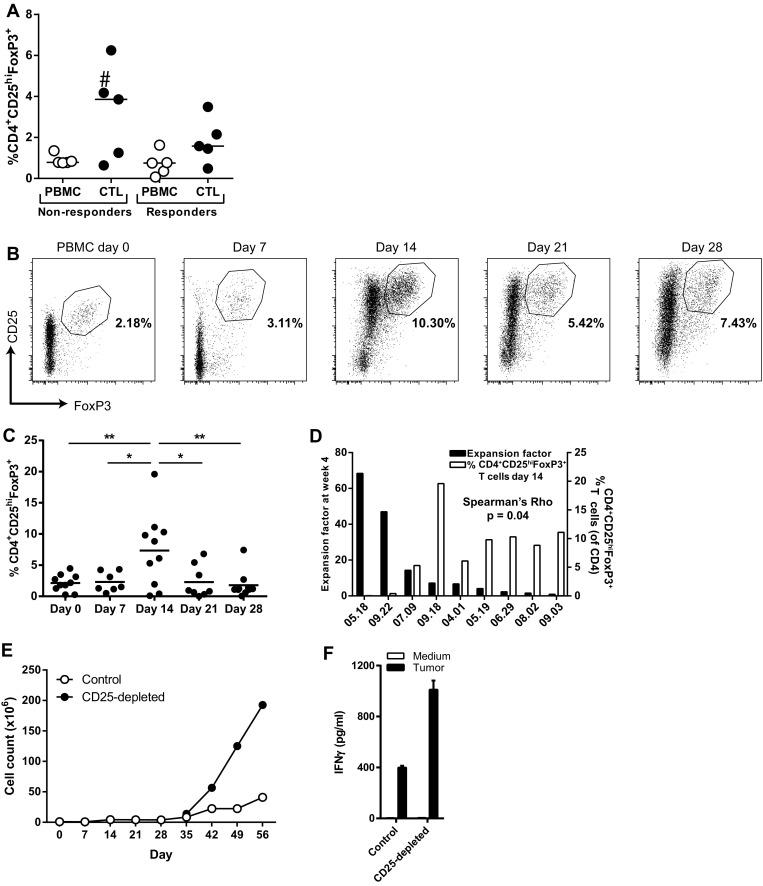
Accumulation of CD4^+^CD25^hi^FoxP3^+^ T cells during MLTC. **a** CD4^+^CD25^hi^FoxP3^+^ T cell frequencies depicted as percentage of CD3^+^ T cells in the PBMC that were used for generation of T cells in the MLTC (PBMC) as well as in the T cell batch that was generated in 4 weeks (CTL). ^#^T cell batch that was used for depletion of CD25^+^ cells in **e. b** Representative plots of the flow cytometric analysis of CD4^+^CD25^hi^FoxP3^+^ T cells during the MLTC. **c** CD4^+^CD25^hi^FoxP3^+^ T cell frequencies (depicted as percentage of CD3^+^ T cells) peak at day 14 during the MLTC. Differences in CD4^+^CD25^hi^FoxP3^+^ T cell frequencies were statistically analyzed by a one-way ANOVA with Tukey post hoc comparisons; *p < 0.05 and **p < 0.01. **d** Expansion factors of T cells generated in different MLTC and the frequencies of CD4^+^CD25^hi^FoxP3^+^ T cells in the same MLTC at day 14. **f** IFNγ concentrations in the culture supernatant after overnight stimulation of the T cell batch with autologous tumor cells. Mean data with standard deviation of one experiment performed in triplo are shown

Tumor-reactive T cell batches were generated in MLTC by weekly stimulation of PBMC with autologous tumor cells. Sufficient cell numbers for infusion could be reached after one MLTC of 4 weeks for some patients, while for others multiple MLTC were needed to reach the required cell numbers for infusion. The expansion rates of T cells were highest in the second half of the MLTC (week 2–week 4). Analysis of the T cell batches that were infused into the patients in our ongoing clinical protocol [5] showed that they contain CD4^+^CD25^hi^FoxP3^+^ T cells (Supplementary Figure S1a). Importantly, while there were no overt differences between the frequencies of CD4^+^CD25^hi^FoxP3^+^ T cells in the PBMC used for MLTC, it became clear that higher frequencies of these cells were observed after the MLTC culture period in T cell batches used for treatment of non-responder patients (Fig. 1a). This suggests that the relatively high frequencies of CD4^+^CD25^hi^FoxP3^+^ T cells observed in 3 out of 5 infusion products from non-responders accumulated during culture. Subsequently, the expansion of CD4^+^CD25^hi^FoxP3^+^ T cells was analyzed during the MLTC cultures. There was a peak in CD4^+^CD25^hi^FoxP3^+^ T cells frequency at day 14 of the MLTC (Fig. 1b, c), and there was a direct inverse correlation between CD4^+^CD25^hi^FoxP3^+^ T cell frequencies and the final expansion of T cells at the end of the MLTC (Spearman’s rho, *r* = −0.700, *p* = 0.04) (Fig. 1d). Since we previously found that the expansion of tumor-specific T cells became visible after 2 weeks of culture, our data suggested that the presence of high numbers of CD4^+^CD25^hi^FoxP3^+^ T cells at this time point had a negative impact on overall T cell proliferation.

**Fig. 2:**
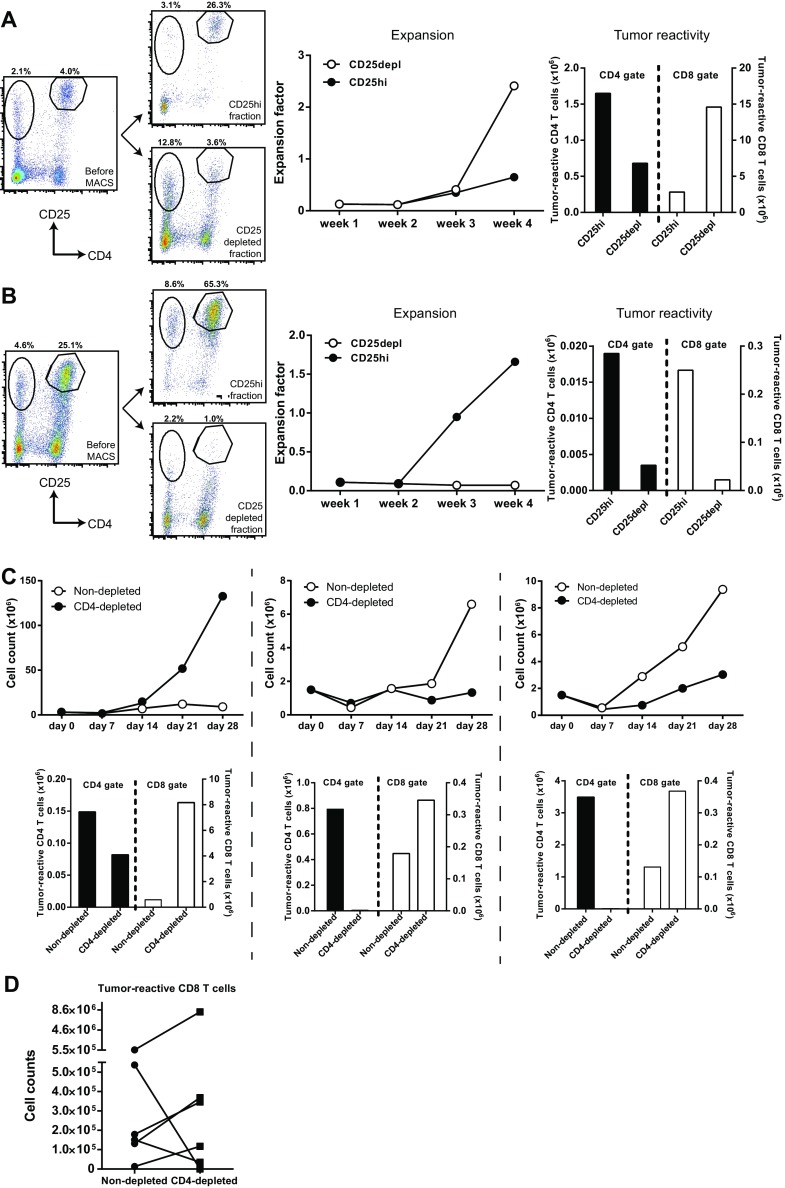
Depletion strategies to eliminate CD4^+^CD25^hi^FoxP3^+^ T cells from MLTC. **a, b** CD4^+^CD25^hi^FoxP3^+^ T cells were depleted at week 2 of MLTC using MACS separation and CD25 microbeads. The T cells were weekly counted to calculate the expansion factor. Tumor reactivity was tested by overnight stimulation of the T cells at week 4 of MLTC with autologous tumor cells. CD137 expression was used to identify the tumor-reactive CD4^+^ and CD8^+^ T cells. The absolute number of tumor-reactive T cells was calculated with the expansion factor of the MLTC. **a** Example of CD4^+^CD25^hi^FoxP3^+^ T cell depletion in which specifically the CD25^hi^ T cells were depleted (*left panel*), resulting in increased expansion of T cells in the MLTC (*middle panel*) and increased numbers of CD8^+^ tumor-reactive T cells (*right panel*). **b** Example of depletion in which not only the CD25^hi^ T cells but also the CD25^+^ activated T cells were depleted from the MLTC (*left panel*), resulting in decreased expansion of T cells (*middle panel*) and decreased numbers of tumor-reactive T cells (*right panel*). **c** Expansion factors (*upper row*) and numbers of tumor-reactive T cells (*lower row*) in MLTC with CD4-depleted PBMC. **d** The number of tumor-reactive CD8^+^ T cells at week 4 of 6 independent MLTC that were started with CD4-depleted PBMC compared to non-depleted PBMC (control)

To functionally assess if the co-expanded CD4^+^CD25^hi^FoxP3^+^ T cells were responsible for the low expansion capacity of tumor-specific T cells, we first depleted the CD4^+^CD25^hi^FoxP3^+^ T cells from a T cell infusion product from a non-responder patient with a high CD4^+^CD25^hi^FoxP3^+^ T cell frequency and compared the proliferation of the CD4^+^CD25^hi^FoxP3^+^ T cell-depleted T cell batch to the non-CD4^+^CD25^hi^FoxP3^+^ T cell-depleted T cells during MLTC. CD4^+^CD25^hi^FoxP3^+^ T cell depletion resulted in increased proliferation (Fig. 1e) and tumor-specific IFNγ secretion (Fig. 1f) of the T cells, indicating that the CD4^+^CD25^hi^FoxP3^+^ T cells were capable of suppressing the expansion of tumor-reactive T cells. Based on this result, a series of experiments was performed to improve the expansion of T cells in the MLTC by elimination of CD4^+^CD25^hi^FoxP3^+^ T cells at week 2 of the MLTC using a GMP-compliant MACS procedure for CD25-depletion. Only when this procedure resulted in the selective depletion of CD25^hi^ cells (Fig. 2a, left panel), it was associated with an improved expansion of T cells (Fig. 2a, middle panel), and an increased number of CD8^+^ tumor-reactive cells (Fig. 2a, right panel), similar to our initial experiment. However, in cases where this method not only led to the depletion of CD25^hi^ T cells but also in that of CD25^+^ effector T cells (Fig. 2b, left panel), the CD25-depleted fraction hardly expanded and lower numbers of CD8^+^ tumor-reactive cells were detected (Fig. 2b, middle and right panel). We repeated this protocol eight times using autologous PBMC and tumor cells from several patients but the variability in outcome remained and apparently was associated with the quality of the separation between CD25^hi^ T cells and CD25^+^ T effector cells. If only CD25^hi^ T cells and not CD25^+^ T effector cells were depleted, it resulted in an improved expansion of tumor-reactive T cells in three out of four experiments (Supplementary Figure S1b). As an alternative for the depletion of CD4^+^CD25^hi^FoxP3^+^ T cells, we depleted the complete CD4^+^ fraction from the PBMC at the start of the MLTC (Supplementary Figure S2). CD4^+^ T cell depletion was very consistent and almost complete, although low numbers of CD4^+^ T cells were still detectable during the culture. Although CD4^+^ T cell depletion did not always result in increased expansion rates of the T cells (Fig. 2c, upper panels), the number of tumor-reactive CD8^+^ T cells was often increased (Fig. 2c, lower panels). In four out of six MLTC using the PBMC and melanoma cells of different patients, CD4^+^ T cell depletion increased the absolute number of tumor-reactive CD8^+^ T cells obtained after 4 weeks of MLTC. However, the opposite effect was observed for the two other MLTC (Fig. 2d). Hence, also CD4^+^ T cell depletion does not guarantee a more pronounced expansion of tumor-specific CD8^+^ T cells.

In conclusion, the presence of high percentages of CD4^+^CD25^hi^FoxP3^+^ T cells in the MLTC cultures suppresses the capacity of tumor-reactive T cells to proliferate upon stimulation with autologous tumor cells, but the specific depletion of CD4^+^CD25^hi^FoxP3^+^ T cells using fully GMP-compliant procedures and CD25-specific or CD4-specific antibodies is not robust enough to guarantee good tumor-reactive T cell expansions during MLTC.

### Tumor cell intrinsic factors inhibit the activation and proliferation of PBMC

**Fig. 3:**
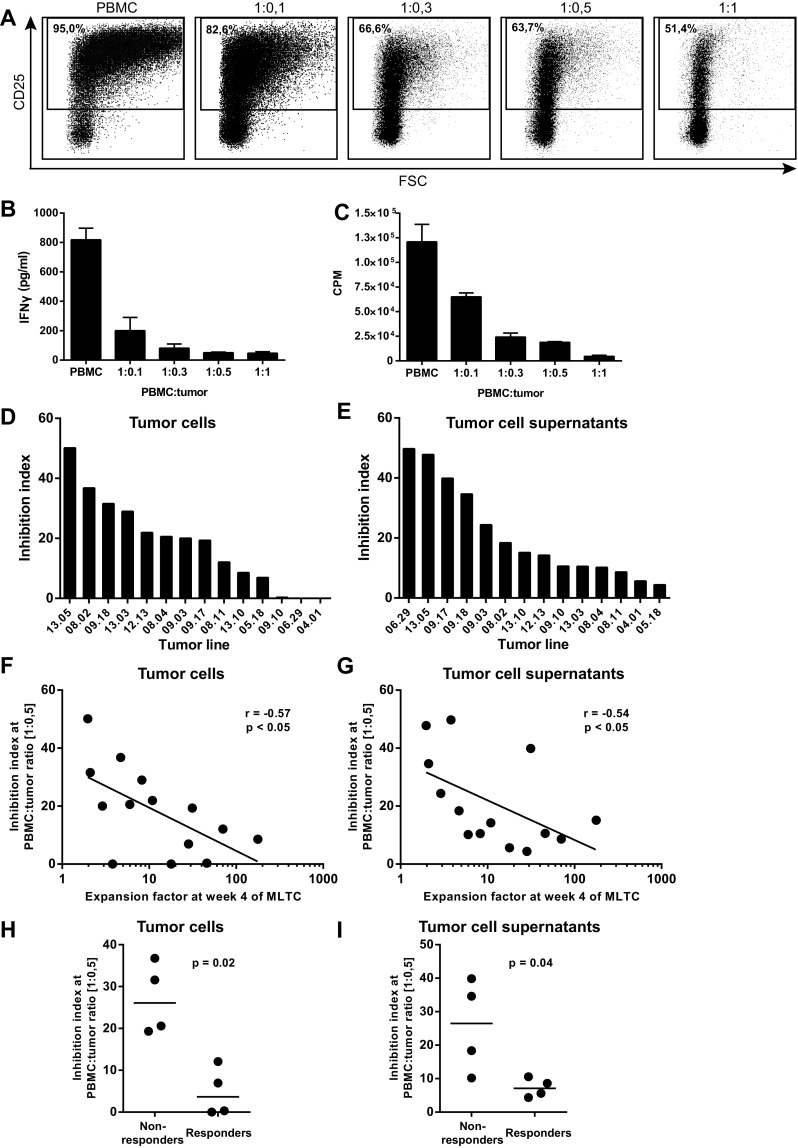
Tumor cells show inhibitory capacity in short inhibition assay. **a** Flow cytometric analysis of CD25 expression on T cells within PBMC stimulated with PHA for 48 h at different ratios of PBMC:tumor cells ratios. **b** IFNγ concentrations present in the cell culture supernatant of the short inhibition assay. **c** Proliferation as measured by ^3^[H]-thymidine incorporation after 5 days of culture in the short inhibition assay. Inhibition index at the 1:0.5 PBMC to tumor cell ratio (**d**) or in presence of tumor cell supernatants (**e**). **f, g** Correlation between inhibition index at 1:0.5 PBMC to tumor cell ratio and the expansion factors at week 4 of the MLTC, using Spearman’s rho correlation. **h, i** The inhibition index at 1:0.5 PBMC to tumor cell ratio for the clinical responder and non-responder patients. Differences were statistically analyzed with the non-parametric Mann–Whitney *U* test. Inhibition index = 100 − (%CD25^+^ [PBMC:tumor]/%CD25^+^ [PBMC] × 100)

To examine the T cell suppressive capacity of melanoma tumor cell lines, we set up a short assay in which we examined the activation of T cells in the presence of increasing numbers of tumor cells. When we used tumor cells derived from a patient whose PBMC displayed a low expansion capacity in the MLTC, we observed decreased expression of the activation marker CD25 on T cells (Fig. 3a), lower concentrations of IFNγ in the culture supernatant (Fig. 3b) and decreased proliferation of PBMC with increasing tumor cell concentrations (Fig. 3c). A large variation was observed between different tumor cell lines in their capacity to inhibit T cell activation (Fig. 3d). To test whether the inhibition exerted by tumor cells was contact dependent, we repeated the experiments with tumor cell supernatants (TSN) showing that T cell activation was also inhibited by TSN. Although the inhibitory capacity varied between the TSN of different tumor cell lines (Fig. 3e), our results indicate that tumor-induced inhibition of T cell activation is dependent on soluble factors.

To analyze the predictive value of the short inhibition assay for the capacity of a tumor cell line to effectively induce T cell expansion in the MLTC, we plotted the inhibitory capacity against the expansion index at week 4 of the MLTC (Fig. 3f, g). A negative correlation exists between the inhibitory capacity and the expansion of T cells in the MLTC, irrespective of whether inhibition was caused by the tumor cells or TSN (Spearman’s rho, *r* = −0.57, *p* < 0.05 and *r* = −0.54, *p* < 0.05 respectively). Interestingly, when we compared the inhibitory capacity of the tumor cells and TSN of the responder patients and the non-responder patients, it was clear that the tumor cells that were derived from non-responder patients showed a much higher inhibitory capacity than those derived from responder patients (Fig. 3h, i). The inconsistency between the experiments performed with tumor cells and with TSN might be the result of differences in metabolic activity of tumor cells during the experiments, which could be the result of differences in cell–cell interactions between the two conditions. Unfortunately, we could not include all ten patients (five responders and five non-responders) in these experiments, since not all tumor cells were available in sufficient quantities. These data not only indicate that the outcomes of the short inhibition assay reflect the ability of tumor cells to induce expansion of tumor-specific T cells in a MLTC but also that tumor cells suppress the activation and expansion of autologous tumor-specific T cells via soluble mediators.

### Inhibition of IDO increases the activation and expansion of tumor-specific T cells in a MLTC

**Fig. 4:**
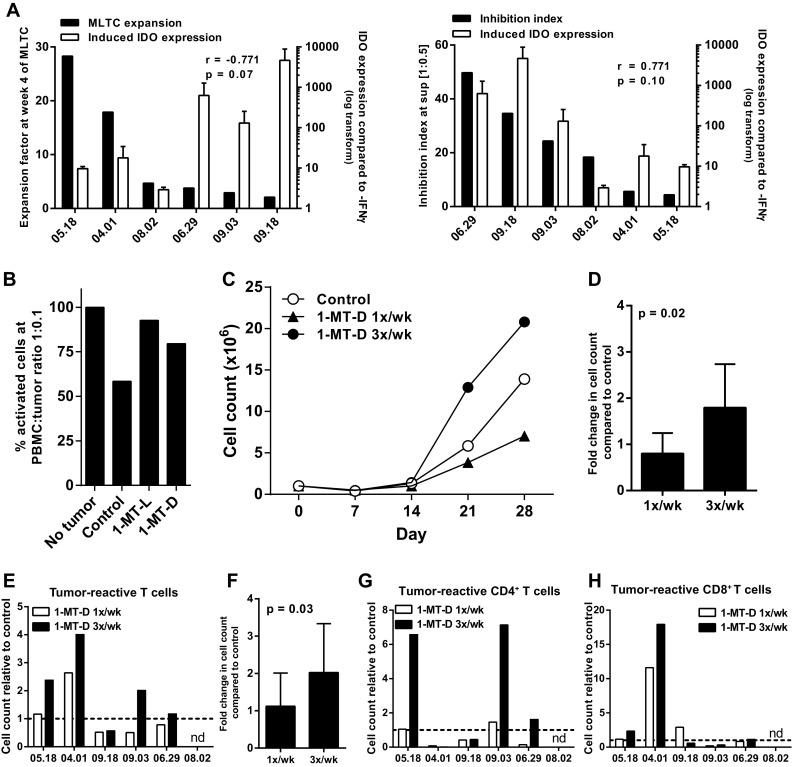
Inhibition of IDO expansion potential in MLTC. **a** IFNγ-induced mRNA expression of IDO by tumor cell lines was assessed by qPCR. IDO expression was plotted together with the expansion factor of the T cells in the MLTC with the same tumor cells (*left panel*) as well as with the inhibition index of the tumor cell line in the short inhibition assay at a 1:0.5 PBMC to tumor cell ratio (*right panel*). The correlation was calculated with Spearman’s rho correlation analysis. **b** The percentage of CD25^+^ activated T cells when two isoforms of the IDO inhibitor 1-MT were added to the short inhibition assay. **c** T cell counts of the MLTC to which 1-MT-D was added once or three times per week. **d** Increase in cell counts at week 4 of the MLTC after addition of 1-MT-D once or three times per week compared to without addition of 1-MT-D. Mean relative cell counts with SD for six different tumor cell lines (student’s *T* test). **e** Increase in the number of CD137^+^ expressing tumor-reactive T cells after overnight stimulation with the autologous tumor cell line at week 4 of the MLTC performed with addition of 1-MT-D, either once or three times per week. Tumor line 08.02 performed so badly in the MLTC that not enough T cell were generated to perform the reactivity test. **f** Fold-change in tumor-reactive CD3^+^CD137^+^ T cell counts at week 4 of the MLTC with1-MT-D once or three times per week over no 1-MT-D control. Mean relative cell counts with SD for five different tumor cell lines (student’s *T* test). The increase in tumor-reactive (CD137^+^) cell counts as shown in (E) for CD4^+^ T cells (**g**) and CD8^+^ T cells (**h**)

One of the mechanisms underlying the suppression of T cell activation and expansion by soluble mediators might be IDO-induced tryptophan starvation [23]. Analysis of IDO mRNA expression by RT-qPCR showed that all cell lines expressed IDO, and that for most cell lines, this expression was increased upon stimulation with IFNγ for 48 h. There were substantial differences in the level of upregulation between the tested tumor cell lines. Importantly, the IFNγ-induced expression of IDO by tumor cell lines was inversely correlated with the expansion rate of T cells in the corresponding MLTC (Fig. 4a, left panel) and directly associated with the capacity of a tumor cell line to inhibit activation of T cells (Fig. 4a, right panel). To test whether IDO was involved in this suppression we used the two isoforms of the IDO inhibitor 1-MT (1-MT-D and 1-MT-L). Both isoforms successfully inhibited IDO activity as measured by a decreased kynurenine concentration (Supplementary Figure S3a) and partly restored the tumor cell induced inhibition of T cell activation (Fig. 4b). Although 1-MT-L had a stronger effect on the kynurenine levels than 1-MT-D, our data suggest that the latter was better with respect to the expansion of activated T cells in the MLTC (Supplementary Figure S3b). To compensate for the lower anti-kynurenine effect of 1-MT-D, we added this compound three times per week to the MLTC cultures and showed that this was more effective than adding 1-MT-D only once a week (Fig. 4c, d). For five out of six tumor cell lines, the addition of 1-MT-D 3 times per week improved the proliferation rate of the T cell batch, whereas addition of l-tryptophan improved the T cell proliferation in three out of four experiments (Supplementary Figure S3c) and as a result in increased numbers of tumor-reactive T cells for four out of six tumor cell lines (Fig. 4e, f). The increase of tumor-reactive cells was observed in both the CD4^+^ T cell and/or the CD8^+^ T cell populations (Fig. 4g, h). Not only the number of tumor-reactive T cells was increased but also the percentage of polyfunctional T cells producing the inflammatory cytokines IFNγ and IL-2 was increased (Supplementary Figure S3d). We concluded that inhibition of IDO by 1-MT-D can improve the activation and expansion of tumor-reactive T cells against melanoma in MLTC.

### Tumor cell-derived galectin-3 inhibits the activation of T cells

**Fig. 5:**
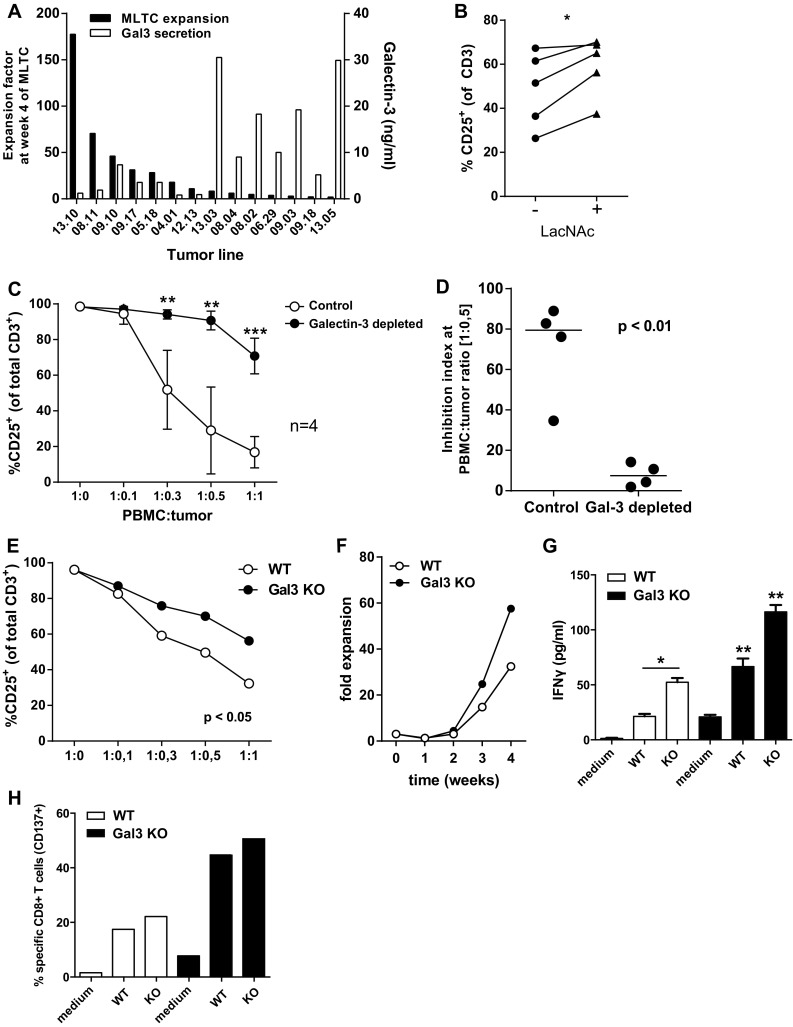
Galectin-3-mediated tumor cell induced T cell inhibition. **a** Comparison of the expansion factor of T cells at week 4 of the MLTC and the galectin-3 secretion in 48 h supernatant of the tumor cell lines used. **b** The percentage of CD25^+^ activated T cells in the short inhibition assay with or without the galectin-3 inhibitor LacNAc (5 mM). The difference between the conditions was analyzed using a one-way ANOVA with a Tukey post hoc test. **c** CD25^+^ activated T cells in the short inhibition assay performed with galectin-3 depleted TSN compared to non-depleted control TSN. Mean with SD for 4 different tumor cell lines (two-way ANOVA with multiple comparisons; **p < 0.01 and ***p < 0.001). **d** The inhibition index at a 1:0.5 PBMC to tumor cell ratio of the control condition compared to the galectin-3-depleted TSN (unpaired *T* test). **e** CD25^+^ activated T cells in the short inhibition assay performed with a galectin-3 knout-out tumor cell line compared to the wild-type tumor cell line (paired *T* test). **f** Expansion factors of T cells that were induced in an MLTC with autologous wild-type tumor cells and galectin-3 knock-out tumor cells. **g** IFNγ concentrations in the culture supernatant after overnight stimulation with autologous tumor cells of the T cell batches generated against the wild-type melanoma cells (*white bars*) and against the galectin-3 knock-out melanoma cells (*black bars*). T cell was stimulated with both the wild-type and galectin-3 knock-out tumor cells, as depicted on the *x-axis*. **h** Frequencies of tumor-specific (CD137^+^) CD8^+^ T cells in T cell batch after overnight stimulation with autologous tumor cells

The soluble factor galectin-3 might play a role by tumor-induced suppression of T cell activation. Analysis of galectin-3 secretion by ELISA showed that most tested tumor cell lines produced galectin-3 to variable amounts (Fig. 5a). The level of galectin-3 secretion was negatively correlated with the final expansion factor of the T cells at the end of the MLTC performed with these tumor cells (Fig. 5a). To study whether galectin-3 inhibited T cell activation, the lectin-inhibitor LacNAc was added in the short inhibition assay. This significantly reversed the tumor-induced inhibition of T cell activation (Fig. 5b), but the effects were not dramatic which might be attributed to the fact that LacNAc itself also hampers T cell activation (Supplementary Figure S4). In addition, LacNAc can also inhibit other galectins, including the immunosuppressive galectin-1. To specifically address the role of galectin-3 and to prevent the intrinsic immunomodulating activity of LacNAc, we depleted galectin-3 from the TSN and compared T cell activation in cultures where either galectin-3 depleted or control TSN was used. Depletion of galectin-3 almost completely alleviated the inhibition of T cell activation by TSN (Fig. 5c, d). Finally, we knocked out galectin-3 from a tumor cell line using CRISPR/Cas9 technology. Again, less inhibition of T cell activation was found for the cell line lacking galectin-3 (Fig. 5e). To validate these findings, we used the galectin-3 knock-out cell line to induce tumor-reactive T cells in a MLTC, and this resulted in a marked increase in the expansion of T cells (Fig. 5f), and also a significant increase of IFNγ secretion by tumor-reactive T cells after stimulation with the autologous tumor cells (Fig. 5g) and an increased frequency of tumor-specific CD8^+^ T cells (Fig. 5h).

## Discussion

Mixed lymphocyte tumor cell cultures (MLTC) can be very efficient for the generation of high numbers of tumor-specific T cells to treat melanoma patients with adoptive cell transfer [5, 6, 33]. In selected cultures, these T cell batches were shown to contain high frequencies of neoepitope-specific T cells, selectively recognizing autologous tumor cells but not HLA-matched melanoma cell lines or known shared antigens [6, 7]. However, not for every patient the expansion of tumor-specific T cells using MLTC is successful and our experiments show that at least three factors, i.e., increased frequencies of CD4^+^CD25^hi^FoxP3^+^ T cells, the production of IDO, and the production of galectin-3 by the tumor cells, play a role in this hampered expansion of tumor-specific T cells.

We found that the presence of CD4^+^CD25^hi^FoxP3^+^ T cells, which accumulate during culture, can suppress the expansion of tumor-reactive T cells. A clear induction of CD4^+^CD25^hi^FoxP3^+^ T cells during culture with a peak at week 2 was associated with impaired expansion of the T cell batch and, therefore, we chose to deplete these culture-induced CD4^+^CD25^hi^FoxP3^+^ T cells, a population which may contain both activated effector T cells and suppressive T cells, at this point of the MLTC. However, MACS technology, which is the only GMP-grade technology that is currently available for separating cell populations, was not sensitive enough to distinguish between CD25^+^effector T cells and the immunosuppressive cells that are more abundant in the CD25^hi^ population. Different results might be obtained when CD4^+^CD25^hi^FoxP3^+^ T cells are depleted at the start of the culture, as was shown by Li et al. [27]. Since we had no evidence for the presence of large numbers of CD4^+^CD25^hi^FoxP3^+^ T cells in PBMC at the start of the culture, we did not perform depletion studies at this point. However, the observation that depletion of CD4^+^ T cells at the start of the MLTC enhanced the generation of tumor-specific CD8^+^ T cells indicates that prevention of CD4^+^CD25^hi^FoxP3^+^ T cell accumulation is beneficial for an efficient MLTC. However, in view of the fact that CD4^+^ T cells play a crucial role in optimal tumor-specific CTL activity [34, 35] and are themselves capable of recognizing and controlling melanoma [6, 36–39], it seems unwise to prevent potential CD4^+^CD25^hi^FoxP3^+^ T cell accumulation at the cost of tumor-specific CD4^+^ T cells.

To investigate the role of immunomodulatory molecules expressed by the tumor cells, we used a short inhibition assay of which we showed that it could predict the expansion of tumor-specific T cells in a MLTC. We showed that IDO negatively affects the expansion of tumor-reactive T cells and that this could be reversed by the IDO inhibitor 1-MT-D and to a lesser extent by 1-MT-L. To unravel whether the suppressive effect of IDO is mediated via depletion of tryptophan or formation of toxic metabolites like kynurenin, the effect of tryptophan addition was studied. In contrast to IDO inhibition by 1-MT-D, the addition of tryptophan did not improve expansion of tumor-specific T cells in all cases, suggesting that the detrimental effect of IDO on tumor-specific T cell expansion was not merely mediated by tryptophan depletion. In melanoma, the expression of IDO by the tumor has been associated with impaired survival [10] and expression of IDO by circulating immune cells was described to be increased with advanced disease stage in melanoma patients [40]. The two isomers of 1-MT, 1-MT-D and 1-MT-L, have a potentially different activity which is dependent on the biological context of IDO expression [41–43]. In our MLTC system, 1-MT-D was superior over 1-MT-L. Moreover, 1-MT-D already showed anti-tumor activity in a mouse model of transplantable melanoma [43] and is currently under clinical development for the treatment of different cancer types [41]. Moreover, the development of IDO inhibitors has been placed on the priority list of immunotherapeutic agents by the National Cancer Institute [44], which is important because IDO should also be blocked in vivo where it is locally produced in the tumor-microenvironment.

We also identified galectin-3 as a tumor-derived factor hampering the generation of tumor-reactive T cells in a MLTC. Galectin-3 regulates T cell function by several mechanisms [20], among others by negative regulation of the TCR-mediated T cell response [19]. Interfering with galectin-3 binding to tumor infiltrating lymphocytes (TIL) boosted the cytotoxicity of these TIL [16]. The use of galectin-3-depleted tumor supernatants and the use of a galectin-3 knock-out tumor line enabled us to specifically eliminate tumor-derived galectin-3 in our culture system. Despite the fact that T cells might also produce galectin-3, elimination of tumor-derived galectin-3 resulted in improved expansion of tumor-reactive T cells, indicating an important role for the galectin-3 produced by the tumor in the suppression of T cell activation. Galectin inhibitors are currently developed for clinical application [45, 46], and maybe extremely useful for both in- and ex vivo applications to boost the anti-tumor response, especially since these inhibitors can also abrogate the potential immunosuppressive effect of other galectins (e.g., galectin-1) secreted by tumor cells [47].

We conclude that the induction of CD4^+^CD25^hi^FoxP3^+^ T cells and the expression of IDO and galectin-3 by melanoma cells are undesirable for a good expansion of tumor-reactive T cells in a MLTC. Although CD4^+^CD25^hi^FoxP3^+^ T cell depletion strategies turned out not to be robust enough to allow implementation into a clinical protocol aiming at improved generation of tumor-reactive T cells, inhibition of IDO and galectin-3 proved to be successful. The tumor-derived suppressive mechanisms that we found to be negatively regulating the ex vivo expansion of tumor-reactive T cells are probably not limited to MLTC but hamper T cell reactivity in the tumor-microenvironment in vivo as well. Therefore our findings could have an impact on the therapeutic potential of other adoptive T cell transfer and immunotherapeutic protocols and may lead to a more successful treatment of metastatic melanoma patients.

## Electronic supplementary material

Below is the link to the electronic supplementary material.

Supplementary material 1 (PDF 1746 KB)

## Abbreviations

1-MT: 1-Methyl-tryptophan
Cas: CRISPR associated system
CRISPR: Clustered regularly interspaced short palindromic repeats
GAPDH: Glyceraldehyde 3-phosphate dehydrogenase
LacNAc: Lectin-inhibitor *N*-acetyl-d-lactosamine
LAG-3: Lymphocyte-activation gene 3
MLTC: Mixed lymphocyte tumor culture(s)
RT-qPCR: Quantitative reverse transcription polymerase chain reaction
Treg: Regulatory T cell
TSN: Tumor-derived cell culture supernatants

## References

[1] Rosenberg SA, Yannelli JR, Yang JC, Topalian SL, Schwartzentruber DJ, Weber JS, Parkinson DR, Seipp CA, Einhorn JH, White DE (1994). Treatment of patients with metastatic melanoma with autologous tumor-infiltrating lymphocytes and interleukin 2. J Natl Cancer Inst.

[2] Erdag G, Schaefer JT, Smolkin ME, Deacon DH, Shea SM, Dengel LT, Patterson JW, Slingluff CL (2012). Immunotype and immunohistologic characteristics of tumor-infiltrating immune cells are associated with clinical outcome in metastatic melanoma. Cancer Res.

[3] Kluger HM, Zito CR, Barr ML, Baine MK, Chiang VLS, Sznol M, Rimm DL, Chen L, Jilaveanu LB (2015). Characterization of PD-L1 expression and associated T-cell infiltrates in metastatic melanoma samples from variable anatomic sites. Clin Cancer Res.

[4] Dudley ME, Yang JC, Sherry R, Hughes MS, Royal R, Kammula U, Robbins PF, Huang J, Citrin DE, Leitman SF, Wunderlich J, Restifo NP, Thomasian A, Downey SG, Smith FO, Klapper J, Morton K, Laurencot C, White DE, Rosenberg SA (2008). Adoptive cell therapy for patients with metastatic melanoma: evaluation of intensive myeloablative chemoradiation preparative regimens. J Clin Oncol.

[5] Verdegaal EM, Visser M, Ramwadhdoebe TH, van der Minne CE, van Steijn JA, Kapiteijn E, Haanen JB, van der Burg SH, Nortier JW, Osanto S (2011). Successful treatment of metastatic melanoma by adoptive transfer of blood-derived polyclonal tumor-specific CD4+ and CD8+ T cells in combination with low-dose interferon-alpha. Cancer Immunol Immunother.

[6] Linnemann C, van Buuren MM, Bies L, Verdegaal EM, Schotte R, Calis JJ, Behjati S, Velds A, Hilkmann H, Atmioui DE, Visser M, Stratton MR, Haanen JB, Spits H, van der Burg SH, Schumacher TN (2015). High-throughput epitope discovery reveals frequent recognition of neo-antigens by CD4+ T cells in human melanoma. Nat Med.

[7] Verdegaal EM, de Miranda NF, Visser M, Harryvan T, van Buuren MM, Andersen RS, Hadrup SR, van der Minne CE, Schotte R, Spits H, Haanen JB, Kapiteijn EH, Schumacher TN, van der Burg SH (2016). Neoantigen landscape dynamics during human melanoma-T cell interactions. Nature.

[8] Vereecken P, Debray C, Petein M, Awada A, Lalmand MC, Laporte M, Van Den Heule B, Verhest A, Pochet R, Heenen M (2005). Expression of galectin-3 in primary and metastatic melanoma: immunohistochemical studies on human lesions and nude mice xenograft tumors. Arch Dermatol Res.

[9] Munn DH, Mellor AL (2007). Indoleamine 2,3-dioxygenase and tumor-induced tolerance. J Clin Invest.

[10] Brody JR, Costantino CL, Berger AC, Sato T, Lisanti MP, Yeo CJ, Emmons RV, Witkiewicz AK (2009). Expression of indoleamine 2,3-dioxygenase in metastatic malignant melanoma recruits regulatory T cells to avoid immune detection and affects survival. Cell Cycle.

[11] Ahmadzadeh M, Johnson LA, Heemskerk B, Wunderlich JR, Dudley ME, White DE, Rosenberg SA (2009). Tumor antigen-specific CD8 T cells infiltrating the tumor express high levels of PD-1 and are functionally impaired. Blood.

[12] Braeuer RR, Shoshan E, Kamiya T, Bar-Eli M (2012). The sweet and bitter sides of galectins in melanoma progression. Pigment Cell Melanoma Res.

[13] Liu FT (2005). Regulatory roles of galectins in the immune response. Int Arch Allergy Immunol.

[14] Liu FT, Rabinovich GA (2005). Galectins as modulators of tumour progression. Nat Rev Cancer.

[15] Cedeno-Laurent F, Opperman M, Barthel SR, Kuchroo VK, Dimitroff CJ (2012). Galectin-1 triggers an immunoregulatory signature in Th cells functionally defined by IL-10 expression. J Immunol.

[16] Demotte N, Wieers G, Van Der Smissen P, Moser M, Schmidt C, Thielemans K, Squifflet JL, Weynand B, Carrasco J, Lurquin C, Courtoy PJ, van der Bruggen P (2010). A galectin-3 ligand corrects the impaired function of human CD4 and CD8 tumor-infiltrating lymphocytes and favors tumor rejection in mice. Cancer Res.

[17] Kouo T, Huang L, Pucsek AB, Cao M, Solt S, Armstrong T, Jaffee E (2015). Galectin-3 shapes antitumor immune responses by suppressing CD8+ T cells via LAG-3 and inhibiting expansion of plasmacytoid dendritic cells. Cancer Immunol Res.

[18] Zubieta MR, Furman D, Barrio M, Bravo AI, Domenichini E, Mordoh J (2006). Galectin-3 expression correlates with apoptosis of tumor-associated lymphocytes in human melanoma biopsies. Am J Pathol.

[19] Chen HY, Fermin A, Vardhana S, Weng IC, Lo KF, Chang EY, Maverakis E, Yang RY, Hsu DK, Dustin ML, Liu FT (2009). Galectin-3 negatively regulates TCR-mediated CD4+ T-cell activation at the immunological synapse. Proc Natl Acad Sci USA.

[20] Hsu DK, Chen HY, Liu FT (2009). Galectin-3 regulates T-cell functions. Immunol Rev.

[21] Perillo NL, Pace KE, Seilhamer JJ, Baum LG (1995). Apoptosis of T cells mediated by galectin-1. Nature.

[22] Taylor MW, Feng GS (1991). Relationship between interferon-gamma, indoleamine 2,3-dioxygenase, and tryptophan catabolism. FASEB J.

[23] Munn DH, Zhou M, Attwood JT, Bondarev I, Conway SJ, Marshall B, Brown C, Mellor AL (1998). Prevention of allogeneic fetal rejection by tryptophan catabolism. Science.

[24] Fallarino F, Grohmann U, You S, McGrath BC, Cavener DR, Vacca C, Orabona C, Bianchi R, Belladonna ML, Volpi C, Santamaria P, Fioretti MC, Puccetti P (2006). The combined effects of tryptophan starvation and tryptophan catabolites down-regulate T cell receptor zeta-chain and induce a regulatory phenotype in naive T cells. J Immunol.

[25] Baban B, Chandler PR, Sharma MD, Pihkala J, Koni PA, Munn DH, Mellor AL (2009). IDO activates regulatory T cells and blocks their conversion into Th17-like T cells. J Immunol.

[26] Xu L, Xu W, Jiang Z, Zhang F, Chu Y, Xiong S (2009). Depletion of CD4(+)CD25(high) regulatory T cells from tumor infiltrating lymphocytes predominantly induces Th1 type immune response in vivo which inhibits tumor growth in adoptive immunotherapy. Cancer Biol Ther.

[27] Li Y, Yee C (2008). IL-21 mediated Foxp3 suppression leads to enhanced generation of antigen-specific CD8+ cytotoxic T lymphocytes. Blood.

[28] Danke NA, Koelle DM, Yee C, Beheray S, Kwok WW (2004). Autoreactive T cells in healthy individuals. J Immunol.

[29] Wang HY, Peng G, Guo Z, Shevach EM, Wang RF (2005). Recognition of a new ARTC1 peptide ligand uniquely expressed in tumor cells by antigen-specific CD4+ regulatory T cells. J Immunol.

[30] Wang HY, Lee DA, Peng G, Guo Z, Li Y, Kiniwa Y, Shevach EM, Wang RF (2004). Tumor-specific human CD4+ regulatory T cells and their ligands: implications for immunotherapy. Immunity.

[31] Herin M, Lemoine C, Weynants P, Vessiere F, Van Pel A, Knuth A, Devos R, Boon T (1987). Production of stable cytolytic T-cell clones directed against autologous human melanoma. Int J Cancer.

[32] Braun D, Longman RS, Albert ML (2005). A two-step induction of indoleamine 2,3 dioxygenase (IDO) activity during dendritic-cell maturation. Blood.

[33] Munn DH, Sharma MD, Lee JR, Jhaver KG, Johnson TS, Keskin DB, Marshall B, Chandler P, Antonia SJ, Burgess R, Slingluff CL, Mellor AL (2002). Potential regulatory function of human dendritic cells expressing indoleamine 2,3-dioxygenase. Science.

[34] Wong SB, Bos R, Sherman LA (2008). Tumor-specific CD4+ T cells render the tumor environment permissive for infiltration by low-avidity CD8+ T cells. J Immunol.

[35] Bos R, Sherman LA (2010). CD4+ T-cell help in the tumor milieu is required for recruitment and cytolytic function of CD8+ T lymphocytes. Cancer Res.

[36] Quezada SA, Simpson TR, Peggs KS, Merghoub T, Vider J, Fan X, Blasberg R, Yagita H, Muranski P, Antony PA, Restifo NP, Allison JP (2010). Tumor-reactive CD4+ T cells develop cytotoxic activity and eradicate large established melanoma after transfer into lymphopenic hosts. J Exp Med.

[37] Friedman KM, Prieto PA, Devillier LE, Gross CA, Yang JC, Wunderlich JR, Rosenberg SA, Dudley ME (2012). Tumor-specific CD4+ melanoma tumor-infiltrating lymphocytes. J Immunother.

[38] Kitano S, Tsuji T, Liu C, Hirschhorn-Cymerman D, Kyi C, Mu Z, Allison JP, Gnjatic S, Yuan JD, Wolchok JD (2013). Enhancement of tumor-reactive cytotoxic CD4+ T cell responses after ipilimumab treatment in four advanced melanoma patients. Cancer Immunol Res.

[39] Wang RF, Wang X, Rosenberg SA (1999). Identification of a novel major histocompatibility complex class II-restricted tumor antigen resulting from a chromosomal rearrangement recognized by CD4(+) T cells. J Exp Med.

[40] Chevolet I, Speeckaert R, Schreuer M, Neyns B, Krysko O, Bachert C, Hennart B, Allorge D, van Geel N, Van Gele M, Brochez L (2015). Characterization of the immune network of IDO, tryptophan metabolism, PD-L1, and in circulating immune cells in melanoma. Oncoimmunology.

[41] Moon YW, Hajjar J, Hwu P, Naing A (2015). Targeting the indoleamine 2,3-dioxygenase pathway in cancer. J Immunother Cancer.

[42] Lob S, Konigsrainer A, Schafer R, Rammensee HG, Opelz G, Terness P (2008). Levo- but not dextro-1-methyl tryptophan abrogates the IDO activity of human dendritic cells. Blood.

[43] Hou DY, Muller AJ, Sharma MD, DuHadaway J, Banerjee T, Johnson M, Mellor AL, Prendergast GC, Munn DH (2007). Inhibition of indoleamine 2,3-dioxygenase in dendritic cells by stereoisomers of 1-methyl-tryptophan correlates with antitumor responses. Cancer Res.

[44] Cheever MA (2008). Twelve immunotherapy drugs that could cure cancers. Immunol Rev.

[45] http://www.galectintherapeutics.com. Accessed 20 Dec 2016

[46] Harrison SA, Marri SR, Chalasani N, Kohli R, Aronstein W, Thompson GA, Irish W, Miles MV, Xanthakos SA, Lawitz E, Noureddin M, Schiano TD, Siddiqui M, Sanyal A, Neuschwander-Tetri BA, Traber PG (2016). Randomised clinical study: GR-MD-02, a galectin-3 inhibitor, vs. placebo in patients having non-alcoholic steatohepatitis with advanced fibrosis. Aliment Pharmacol Ther.

[47] Rubinstein N, Alvarez M, Zwirner NW, Toscano MA, Ilarregui JM, Bravo A, Mordoh J, Fainboim L, Podhajcer OL, Rabinovich GA (2004). Targeted inhibition of galectin-1 gene expression in tumor cells results in heightened T cell-mediated rejection; a potential mechanism of tumor-immune privilege. Cancer Cell.

