# Supplementary material for: IDO and galectin-3 hamper the ex vivo generation of clinical grade tumor-specific T cells for adoptive cell therapy in metastatic melanoma
Source: Cancer Immunol Immunother. 2017 Apr 11;66(7):913–26. doi: 10.1007/s00262-017-1995-x (PMC5489610; doi:10.1007/s00262-017-1995-x)
Supplement: Supplementary file 1 — Supplementary material 1 (PDF 1746 KB) [file 262_2017_1995_MOESM1_ESM.pdf]

Supplementary Figure S1

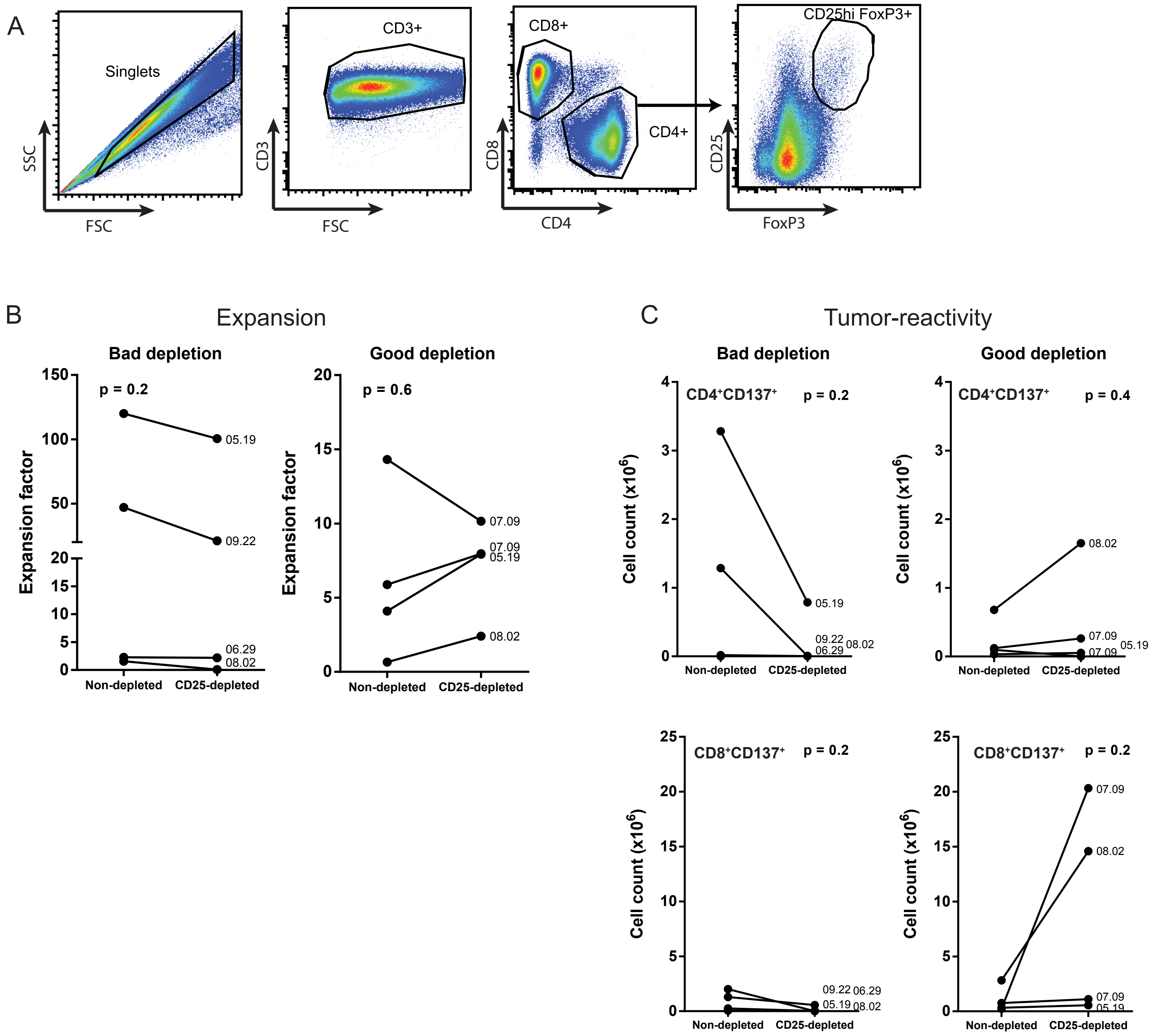

Supplementary Figure S1. The effects of $CD25^{+}$ T cell depletion during MLTC. **(A)** Gating strategy of flow cytometric analysis of $CD4^{+}CD25^{hi}FoxP3^{+}$ T cells in PBMC and T cell batches. **(B,C)** Expansion factors **(B)** and numbers of tumor-reactive T cells **(C)** of non-depleted and CD25-depleted T cell batches divided for experiments with bad and good depletion of Tregs after MACS separation with CD25-specific beads.

Supplementary Figure S2

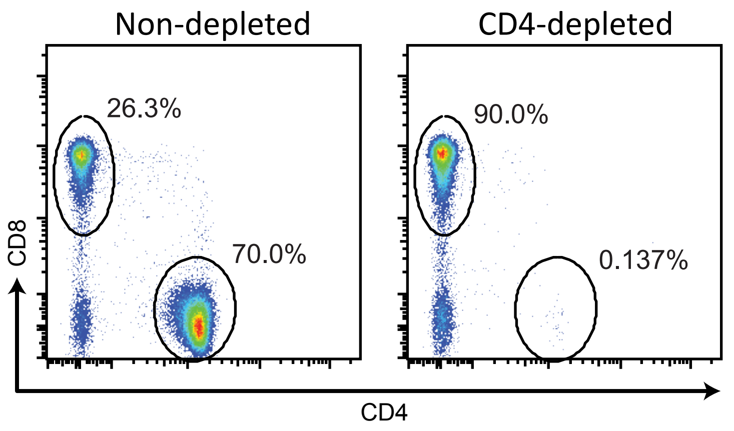

Supplementary Figure S2. Efficacy of CD4 depletion in PBMC.

Flow cytometric analysis of PBMC that were depleted for CD4 T cells using MACS technology.

Supplementary Figure S3

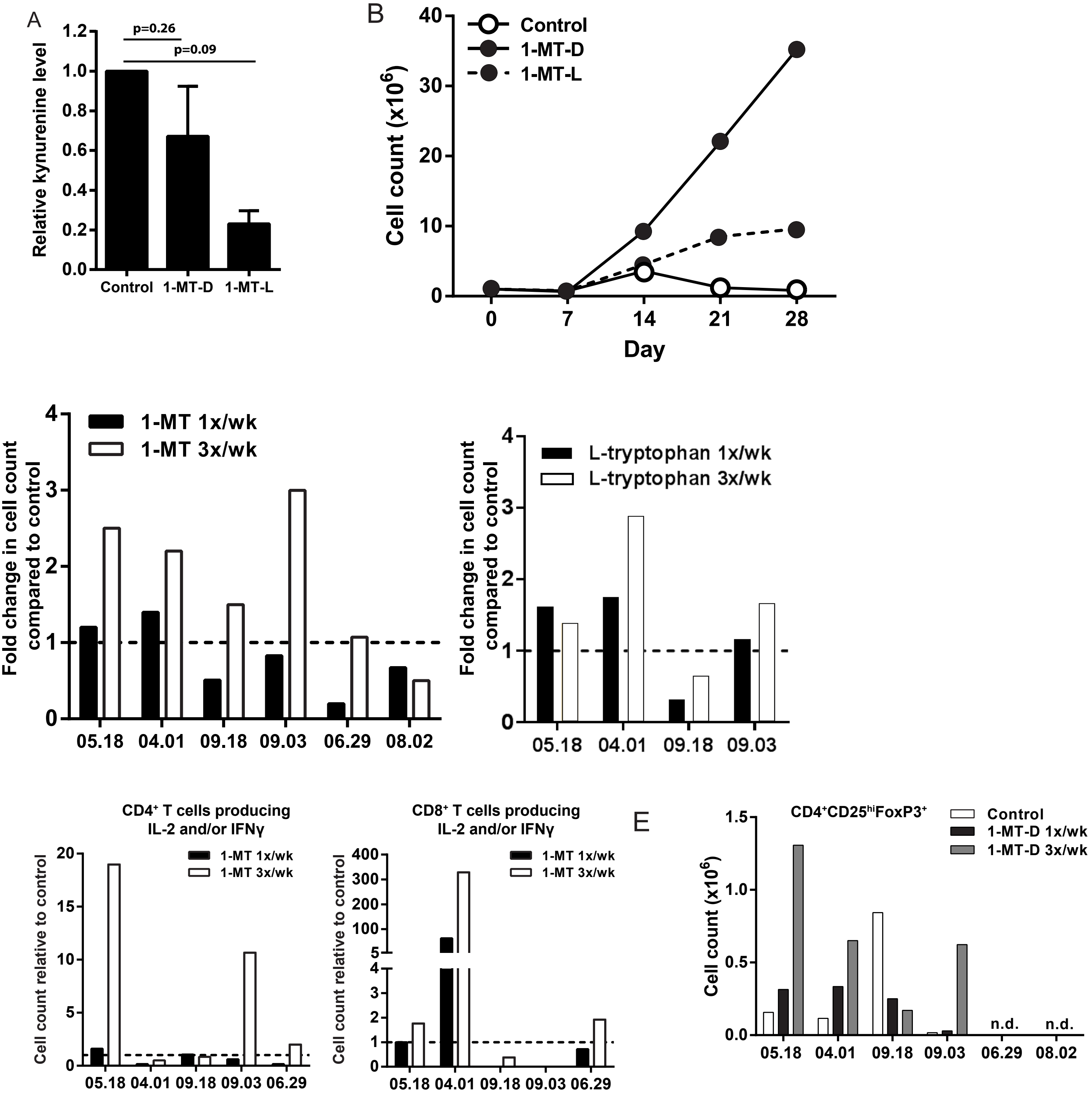

Supplementary Figure S3. The effects of IDO inhibition during MLTC.

(A) The relative kynurenine concentrations in the culture supernatant of the MLTC after addition of the IDO inhibitors 1-MT-D and 1-MT-L. **(B)** Expansion of T cells in MLTC to which either 1-MT-D or 1-MT-L was added once a week. **(C)** Fold change in cell counts compared to control at week 4 of MLTC of 6 independent experiments comparing the addition of 1-MT-D once a week versus three times per week (left panel) and addition of L-tryptophan once a week versus three times a week (right panel) (fold change compared to control culture without addition of 1-MT-D). **(D)** Fold change in cell counts of cytokine (IL-2 and/or IFNγ) producing tumor-reactive ($CD137^{+}$) cells compared to control at week 4 of MLTC of 5 independent experiments comparing the addition of 1-MT-D once a week versus three times per week (fold change compared to control culture without addition of 1-MT-D). **(E)** Frequencies of Tregs in T cell batches that were generated using MLTC with and without the addition of 1-MT-D.

Supplementary Figure S4

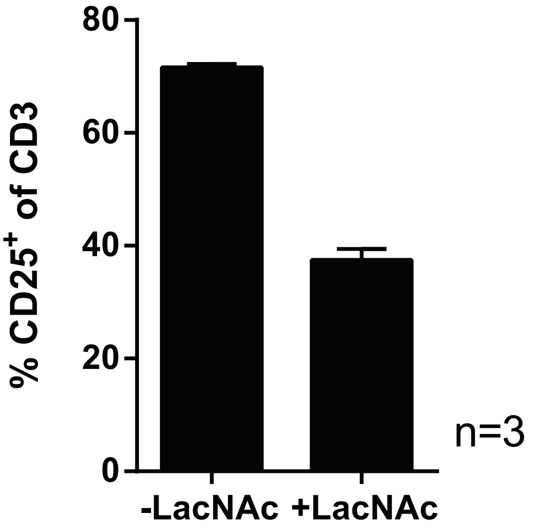

Supplementary Figure S4. The effect of LacNAc on PHA activated CD25$^+$ T cells.

Frequencies of CD25$^+$ cells in the short inhibition assay in the control condition (no tumor cells, only PHA stimulation) with and without addition of LacNAc.
